# Accounting and the US cannabis industry: federal financial regulations and the perspectives of Certified Public Accountants and cannabis businesses owners

**DOI:** 10.1186/s42238-020-00049-7

**Published:** 2020-12-03

**Authors:** G. Suzanne Owens-Ott

**Affiliations:** Colorado Mesa University, 1100 North Avenue, Grand Junction, CO 81507 USA

**Keywords:** Cannabis, Marijuana, Accounting, Business, Certified Public Accountants, Financial regulation, Colorado, Washington State

## Abstract

**Background:**

Cannabis-related businesses (CRBs), in states where cannabis is legal, may be unable to obtain professional financial services including banking, insurance, and accounting because of federal laws and regulations. This qualitative study investigated the following research questions.

1. Why are some Certified Public Accountants unwilling to provide services to cannabis-related businesses?
2. How do CRBs compensate for lack of Certified Public Accountant services?
3. What does a certified public accountant need to know about the cannabis industry prior to engaging to provide services to CRBs?

**Methods:**

Data for this grounded-theory qualitative study was gathered from twenty-three semi-structured phone and face-to-face interviews. Ten cannabis-related business owners were recruited from a convenience sample after attempting a broad recruiting effort. Thirteen Certified Public Accountants with active licenses in Colorado or Washington State participated from firms of varying size and willingness to serve the cannabis industry. The individual interviews, which lasted from twenty minutes to more than an hour, focused on the participants’ perceptions of the complexities of accounting and tax compliance for cannabis businesses.

**Results:**

Eight of the thirteen Certified Public Accountants interviewed would not provide services to the cannabis industry with the primary reason given that cannabis is federally illegal. All ten of the cannabis business owners interviewed indicated they engage a Certified Public Accountant to provide tax services. Seven out of ten CRB participants and ten of the thirteen Certified Public Accountant participants indicated that extensive industry knowledge is needed for an accountant to competently provide services to a CRB.

**Conclusions:**

CRB owners need to carefully consider the industry knowledge and experience of a potential Certified Public Accountant prior to engaging them. This study shows that US Certified Public Accountants should weigh the risk of federal prosecution and potential loss of the Certified Public Accountant license when deciding whether to serve a CRB client. The study also found that a Certified Public Accountant must commit to acquiring and maintaining substantial specialized knowledge related to tax Code Section 280E, internal controls for a cash-only or cash-intensive business, and the workings of the cannabis industry under the current regulatory conditions.

**Supplementary Information:**

**Supplementary information** accompanies this paper at 10.1186/s42238-020-00049-7.

## Background

There are a growing number of cannabis-related businesses (CRBs) in the USA as many states have legalized cannabis for medical and/or recreational use even though it remains illegal at the federal level under the Controlled Substances Act (CSA). Thirty-three states plus the District of Columbia, Puerto Rico, the U.S. Virgin Islands, and Guam have legalized medical cannabis, while eleven states plus the District of Columbia and the Northern Mariana Islands have also legalized recreational cannabis usage as of September 2020 (National Conference of State Legislatures 2020). Table 1 shows the legalized states by category. Cannabis has been on the U.S. Schedule I controlled substances list since 1970 (Campbell 2012). Regardless of state laws, cannabis is a Schedule I drug according to the federal government, and those found trafficking in cannabis could face criminal prosecution (Uniform Controlled Substances Act 1970). The Rohrabacher-Blumenauer amendment prohibits the use of federal funds to prosecute a cannabis-related business activity that operates within “States that have legalized the use of medical marijuana,” and was most recently extended to the 2019–2020 fiscal year in the 2020 Consolidated Appropriations Act (2019).

**Table 1:** States in which cannabis is legal for medical and/or recreational use

States in which cannabis is legal only for medical use	States in which cannabis is legal for medical and recreational use	States in which only CBD (low THC) cannabis is allowed	States in which cannabis remains illegal
AR, AZ, CT, DC, DE, FL, HI, LA, MD, MN, MO, MT, ND, NH, NJ, NM, NY, OH, OK, PA, RI, UT, and WV	AK, CA, CO, IL, MA, ME, MI, NV, OR, VT, and WA	AL, GA, IA, IN, KY, MS, NC, SC, TN, TX, VA, WI, and WY	ID, KS, NE, SD

Abbreviations are standard US Postal Service abbreviations. (National Conference of State Legislatures 2020; Accessed 22 Oct 2020)

Cannabis and hemp are both varieties of the cannabis plant but with different THC content. Hemp products became federally legal with the passage of the Agricultural Improvement Act of 2018 (2018). The bill “removed hemp, defined as cannabis (Cannabis sativa L.) and derivatives of cannabis with extremely low concentrations of the psychoactive compound delta-9-tetrahydrocannabinol (THC) (no more than 0.3 percent THC on a dry weight basis), from the definition of marijuana in the Controlled Substances Act (CSA)” (U.S. Food and Drug Administration 2019). Cannabis remains federally illegal and is the topic of this study.

This conflict between federal and state laws is problematic because cannabis-related businesses and ancillary businesses operating in legalized states and complying with all state laws are unable to fully comply with federal laws. Many cannabis businesses in states where cannabis is legal are facing difficulty in obtaining professional financial services because cannabis is still a controlled substance at the federal level (Taylor et al. 2016). For example, CRB’s face difficulty obtaining banking services such as checking, credit cards, electronic transfers, and loans which results in cash-only or cash-intensive business operation (Taylor et al. 2016). Many Certified Public Accountants may be unwilling to provide their accounting and tax services to CRBs due to increased risks associated with the industry (AICPA 2016; Gottlieb and Munson 2016; Taylor et al. 2016).

Because cannabis is a Schedule I drug, a cannabis business is subject to Internal Revenue Service (IRS) Code Section 280E which disallows the deduction of ordinary business expenses in arriving at taxable income (Internal Revenue Code 1982). Code Section 280E results in significantly higher effective tax rates for cannabis businesses than for other businesses (Alsharaiha 2017; Taillon and Cornelius 2018). The Racketeer Influenced and Corrupt Organizations Act (RICO) of 1970 provides for federal criminal prosecution of individuals involved with criminal activities including drug trafficking (Racketeer Influenced and Corrupt Organizations Act 1970). One does not have to be directly involved with the criminal enterprise to be prosecuted under RICO as it applies to others associated with the activity which could potentially include those providing professional services such as accountants, lawyers, and bankers (US Department of Justice 2009; Reinhart 2016).

Cannabis businesses are also greatly affected by the Bank Secrecy Act (BSA) and anti-money laundering regulations that are designed to help identify and report money laundering activity (Sanders 2015). The Financial Crimes Enforcement Network (FinCEN), a bureau of the U.S. Department of Treasury, exists to “safeguard the financial system from illicit use and combat money laundering” (Financial Crimes Enforcement Network n.d.). In practical terms, FinCEN guidance stated that providing banking services to cannabis businesses was illegal and required banks to self-report this federally illegal activity (Houser and Rosacker 2014; Shu-Acquaye 2016). Due to the BSA risks and FinCEN reporting requirements, most large banks will not provide accounts for cannabis-related businesses (Drayton 2017). However, there is evidence that some banks and credit unions are serving the industry. According to FinCEN, as of June 2020, 695 financial institutions in the USA are reportedly serving the cannabis industry; this is down from a high of 747 institutions in November 2019 (Financial Crimes Enforcement Network [FinCEN], 2020).

Bills have been proposed that would change the federal landscape for the cannabis industry. For example, H.R. 1588, the Ending Federal Marijuana Prohibition Act of 2019 (2019), was introduced in the House of Representatives in March of 2019 and referred to the House Energy and Commerce and House Judiciary committees. There have been several attempts to pass legislation to ease the banking burden for the industry including the Secure and Fair Enforcement (SAFE) Banking Act of 2019 which passed in the House of Representatives, but has not yet passed the Senate (Secure and Fair Enforcement (SAFE) Banking Act of 2019 2019). There have been numerous cannabis-related bills introduced in the 116^th^ Congress in 2019–2020 that range from the MAPLE Act which would remove cannabis from the list of crimes that would prevent an immigrant entrance into the USA to the MORE Act which provides total decriminalization of cannabis and expungement of cannabis-related convictions; however, as of October 2020, none of these bills have passed both chambers (Maintaining Appropriate Protections for Legal Entry (MAPLE) Act of 2019 2019; Marijuana Opportunity Reinvestment and Expungement (MORE) Act of 2019 2019). Changes in federal legislation could potentially reduce, if not eliminate, the business problem studied herein. For example, if federal prohibition were eliminated, cannabis clients would be no different than any other client in a legal industry for a Certified Public Accountant. Passage of a cannabis banking act would allow the industry to utilize regular banking services and eliminate some risk associated with a cash-intensive type of business.

### Accounting and internal control

There are several unique accounting issues that affect the cannabis industry. Due to the state and local regulatory requirements, CRBs need to maintain effective accounting records. Tax Code Section 280E requires attention to inventory accounting while following the tax requirements (Madison 2018). Lack of banking availability requires special attention to internal controls to safeguard cash and maintain good accounting records of cash transactions. State regulations require thorough accounting records as well as strong internal controls (Code of Colorado Regulations 2010/2018; Code of Colorado Regulations 2013/2018; Washington Administrative Code 2013/2016).

Internal controls are the activities that an organization takes to provide reasonable assurance that the company can meet its goals (Frazer 2016; Khairul et al. 2016). Failure to comply with state, local, or tax regulations can result in penalties, fines, and potential revocation of the cannabis license (Hunzicker 2018). Effective internal controls were missing in the *Alterman v. Commissioner* case in which a medical cannabis company failed to maintain adequate records and failed to understand how to comply with Tax Code Section 280E (Lee 2018). Due to complexities in accounting and regulation, cannabis businesses should be perpetually ready for a potential audit by regulators or the IRS (Hunzicker 2018). Certified Public Accountants can provide small businesses, including cannabis businesses, with guidance to develop and implement effective internal controls (Kapp and Heslop 2011).

A cash-intensive business requires some unique internal controls to safeguard the asset and to maintain adequate records of cash transactions (Frazer 2016). The IRS defines a cash-intensive business as one that receives most of its revenues in cash and/or pays many of its expenses in cash (Internal Revenue Service 2010). Cash may be stolen by employees and is most susceptible to theft when entering or exiting the business (Frazer 2016). To maintain proper accounting records to support potential tax and other regulatory audits, a cash-intensive business should “document the flow of each receipt or revenue from the customer’s hands to the business, to the final end in the business bank account or as payment for a business expense” (Internal Revenue Service 2010, p. 7).

Physical controls may be one of the most crucial internal controls, and security must be considered for areas “to include property, office buildings, warehouses, utility rooms … and vehicles as well as employees, contractors, and visitors” (Satnaliwala 2018, p. 22). Physical controls over cash and cannabis inventory include keeping them in a safe or vault, video surveillance, and promptly depositing cash into a bank account if the cannabis business has one (Arens et al. 2017; Code of Colorado Regulations 2013/2018; Simkin et al. 2015). Physical controls should be tested frequently to ensure continuous safeguarding of assets (Satnaliwala 2018). Frequent physical inventory counts are needed and differences between the actual count and inventory records should be examined right away (Kapp and Heslop 2011). The Colorado regulations require daily reconciliation of inventory on hand to the Inventory Tracking System (Code of Colorado Regulations 2010/2018; Code of Colorado Regulations 2013/2018).

### Certified Public Accountant requirements and risks

Certified Public Accountants must adhere to an extremely high standard of ethical conduct as provided by the American Institute of Certified Public Accountants (AICPA) Code of Professional Conduct and individual state codes of conduct (AICPA 2014). Unlike many business professionals, Certified Public Accountants obtain a license or permit to practice from the state(s) in which they provide services (AICPA 2016). A violation of ethics or “lack of good moral character” could potentially result in a loss of the Certified Public Accountant license, and therefore the potential loss of the ability to earn a living (AICPA 2016, p. 11).

Most state boards of accountancy have provided somewhat unclear official guidance on the provision of services for the cannabis industry (AICPA 2016). The Washington State Board of Accountancy (BOA) stated in 2014 and again in 2018 that a Certified Public Accountant’s provision of services to a cannabis-related business does not constitute a violation of the BOA’s rules; this statement followed the March 2018 signing of the Engrossed Substitute Senate Bill 5928 which states that the provision of services to a CRB by a Certified Public Accountant does not, by itself, constitute a crime (Satterlund 2018). The Washington BOA further recommended that Certified Public Accountants consider the risks associated with serving the cannabis industry and that Certified Public Accountants engage an attorney for counsel (Satterlund 2018).

The Colorado BOA issued a Position Statement on December 16, 2015 that indicated the provision of services by a Certified Public Accountant to a cannabis business is not “specifically prohibited by the Accountancy Act” (Colorado Board of Accountancy 2015, para. 1). The Colorado Board went on to caution Certified Public Accountants that their Position Statement does not constitute an endorsement for Certified Public Accountants to enter the industry (Colorado Board of Accountancy 2015).

In January of 2019, the National Association of State Boards of Accountancy (NASBA) in conjunction with the AICPA published a document entitled “Providing services to businesses in the marijuana industry: A sample of current board positions” (NASBA/AICPA 2019). This paper summarized the board positions for Alaska, Arizona, Arkansas, Colorado, Connecticut, Florida, Iowa, Maryland, Massachusetts, Michigan, New Mexico, Nevada, Oregon, and Washington. This sample includes states in which cannabis is legal only for medical purposes, states in which cannabis is legal for recreational or medical usage, and a state in which cannabis remains prohibited. All state boards in the report, except New Mexico, indicated that the Certified Public Accountant would not face disciplinary action by the board for providing services to a cannabis business assuming the Certified Public Accountant was in compliance with all state laws (NASBA/AICPA 2019). This was even the case for Iowa where only CBD (low THC) cannabis is allowed; the Iowa BOA indicated that Iowa Certified Public Accountants serving cannabis businesses in legalized states would not face disciplinary action (NASBA/AICPA 2019).

While providing services to cannabis businesses by itself does not necessarily constitute a violation of good moral character, other problems can result from a Certified Public Accountant serving the cannabis industry (Sterna and Wolfe 2017). A Certified Public Accountant may be found to have “aided and abetted” or been involved in a “conspiracy to violate” the federal Controlled Substances Act or racketeering laws (Sterna and Wolfe 2017, p. 9). A Certified Public Accountant may be exposed to criminal investigation and/or prosecution as well as potential fines, penalties, and sanctions if he participates in “dishonest, fraudulent, or criminal acts” associated with the cannabis industry (Sterna and Wolfe 2017, p. 10).

Cannabis businesses have a higher likelihood of income tax audit than other businesses due to complexities of Tax Code Section 280E (Ohanesian 2018). The large number of cash transactions results in the absence of a paper trail which increases the risk of tax evasion and makes cannabis businesses targets for federal tax audits (Sanders 2015). Some state cannabis regulations including New Mexico, Minnesota, and Colorado may require a financial statement audit of a cannabis business to be completed by a Certified Public Accountant (AICPA 2016; Code of Colorado Regulations 2013/2018).

In addition, Certified Public Accountants may believe there is risk that associating with the cannabis industry could damage their reputation in the business community with current or prospective clients. Reputation of a firm may be seen by outsiders as an indication of the firm’s quality of services (Devers et al. 2009). Certified Public Accounting firms may be viewed as less than legitimate based on their association with the somewhat controversial cannabis industry (Devers et al. 2009). Core-stigmatized organizations are those for whom outsiders have a “perceived violation of social norms” and may be looked at unfavorably (Hudson and Okhuysen 2009, p. 134). Current or prospective clients may avoid associating with a Certified Public Accounting firm who works in the cannabis industry because they worry that negative stigma may transfer to them (Hampel and Tracey 2016). Some Certified Public Accountants may determine that they are willing to accept such core stigma as part of their business strategy as may be the case for Certified Public Accounting firms who specialize in cannabis clients (Hudson and Okhuysen 2009).

Lastly, professional standards require that a Certified Public Accountant only take on engagements for which he or she has the appropriate technical knowledge about the industry to complete the work competently (AICPA 2014). The complexities of the cannabis industry make meeting this standard costly and not necessarily worth the risks to many Certified Public Accountants (AICPA 2016). In general, Certified Public Accountants need to evaluate the many risks associated with providing services to the industry and may determine that the risks outweigh the benefits and choose to not take on cannabis clients (Gaetano 2017).

### Purpose of the study

This qualitative study investigated the following research questions.

1. Why are some Certified Public Accountants unwilling to provide services to cannabis-related businesses?
2. How do CRBs compensate for lack of Certified Public Accountant services?
3. What does a Certified Public Accountant need to know about the cannabis industry prior to engaging to provide services to CRBs?

## Methods

Qualitative research is particularly well-suited for the “early stages of research” as in the case of a young industry such as cannabis (Belotta 2018, p. 2623). The grounded theory approach to qualitative research utilizes “systematical methodological procedures” to identify theories as they “emerge from the data” (Astalin 2013, p. 121). Grounded theory is particularly appropriate for research such as this study which seeks to generate theory based upon the reported data from participants (Savin-Baden and Major 2013). Grounded theory requires one to continuously compare and analyze data previously collected to the new data being collected to identify patterns and themes and produce theories. Interviews of participants are often used as a method of data collection in grounded theory research (Astalin 2013; Cooper and Schindler 2014; Savin-Baden and Major 2013).

Semi-structured interviews of cannabis business owners were used to learn about the financial difficulties CRBs face, particularly related to accounting and finding Certified Public Accountant services. Semi-structured interviews of Certified Public Accountants provided an understanding of the willingness or hesitancy to provide services to CRBs, the potential risks related to the industry, and accounting and tax issues relevant to CRBs. The interview questions for Certified Public Accountants may be found in Additional file 1: Appendix A and for CRB owners may be found in Additional file 2: Appendix B. IRB approval was obtained from California Southern University prior to data collection. Signed statements of informed consent from all participants were emailed or hand delivered during face-to-face interviews. Interviews were one-on-one. Nineteen interviews were conducted via telephone and four conducted in person between February 2019 and May 2019. Interviews lasted from approximately twenty minutes to more than an hour.

### Participants

A list of 3076 active Certified Public Accounting firms was downloaded from the Colorado and Washington State Board of Accountancy websites on December 2 and 3, 2018, respectively. The listed firms’ websites were then reviewed to locate contact information for partners and managers who were emailed a recruitment letter; this was a highly manual process and not all websites had contact information for their employees posted and resulted in less than half the number of email contacts compared to the number of firms. While 1249 requests for participation were emailed, only four willing participants responded. A convenience sample was recruited for the remaining Certified Public Accountant participants by posting requests for participation on LinkedIn and Facebook and through conversations and emails with accounting colleagues in both states. Deliberate effort was made to include participants from a variety of sizes of firms as well as from firms who did serve the cannabis industry and firms that did not. No participants withdrew during the interviews; however, one Certified Public Accountant declined the interview and simply provided a statement that his firm does not provide services to the cannabis industry because it remains federally illegal.

Colorado and Washington State both maintain publicly available listings of licensed cannabis businesses. The Colorado Department of Revenue publishes lists of licensed cannabis facilities on their state website. The listings reported approximately 2200 licensed Colorado facilities for retail stores, retail manufacturing, retail cultivation, and medical cultivation for December 2018. These lists contained only company names, city, zip, and license number. Seventy randomly selected companies from the combined lists were researched on the internet to locate email or phone information. A request for participation was emailed if an email address was listed or if there was a “contact us” option on the website.

The Washington State Liquor and Cannabis Board publishes a report of monthly “Sales Activity by License Number.” For December 2018, 433 cannabis licenses reported sales activity. The 433 license numbers were then cross referenced to the “Washington Listing of Marijuana Applicants” report which provides company names, addresses, and phone numbers. Since phone numbers were readily available on this report, phone calls were made to request participation from randomly selected licensees throughout the list.

Forty-five licensed cannabis businesses were called and seventy were emailed to request owner participation, but finding CRB participants proved to be extremely difficult. No emails were answered. Phone calls never got past the employee answering the phone, meaning the employee indicated they would not be interested or would leave a message for the owner to respond. Only two CRB owners responded, but they both declined participation and generally expressed unease in sharing any information related to their business with a stranger. It became apparent that obtaining participation in this manner was not effective.

Next, requests for participation were posted on LinkedIn and Facebook and requested through conversations with colleagues to identify and recruit potential CRB owners in both states. This led to five leads where there was a personal relationship between someone in my professional network and a CRB owner. One of those five, a small Colorado retailer, did not agree to participate as they were uncomfortable sharing their business information. The other four CRB owners agreed to participate and provided additional names of CRB owners in their networks to contact at the conclusion of their interviews. This process of referrals continued until the study reached ten CRB participants. No participants withdrew during the interviews. The unresponsive CRBs varied in terms of location and type of firm as did those CRBs that participated. Tables 2 and 3 show the descriptive data for the participants.

**Table 2:** Cannabis-related business owners (CRB) participating in interviews

Participant ID^a^	Type of cannabis business	Year established	Legal structure	Approximate 2018 revenues
WACRB1	Producer/Processor	2014	LLC	$3.5M
WACRB2	Producer/Processor	2015	LLC	$600k
WACRB3	Producer/Processor	2015	Partnership	$5k (closing)
WACRB4	Processor	2013	LLC	Declined
WACRB5	Retailer	2017	Partnership	$5.6M
COCRB1	Retailer	2014	LLC	$2.5M
COCRB2	Retailer	2014	LLC	$4M
COCRB3	Producer/Processor	2014	S-Corp	$5–6M
COCRB4	Producer	2018	S-Corp	$0 (start-up)
COCRB5	Retailer	2009 (medical)	LLC	$1M

^a^First two digits of participant ID represent the state, next three digits indicate the participant was a CRB owner, and last digit represents the number of the interviewee

**Table 3:** Certified Public Accountants (CPAs) participating in interviews

Participant ID^a^	Partner or manager within firm	Primary county or US Zip code of practice	Type of firm^b^	Do you or your firm provide services to CRBs?
WACPA1	Partner	98501	Local	Yes
WACPA2	Partner	Various	Single CPA	Yes
WACPA3	Partner	98004	Local	Yes
COCPA1	Partner	81501	Single CPA	No
COCPA2	Partner	80237	National	No
COCPA3	Partner	Nation-wide	National	Yes
COCPA4	Manager	81501	National	No
COCPA5	Partner	81501	Regional	No
COCPA6	Partner	80433	Local	No
COCPA7	Partner	80433 and 81501	Local	No
COCPA8	Partner	80901	Single CPA	Yes
COCPA9	Partner	Denver	Regional	No
COCPA10	Manager	81521	Local	No
COCPA11^c^	Partner	Nation-wide	Big 4	No

^a^First two digits of participant ID represent the state, second three digits indicate the participant was a Certified Public Accountant, and last digit represents the number of the interviewee

^b^Choices were: single Certified Public Accountant (CPA); local CPA firm with multiple CPAs; regional CPA firm; national CPA firm; Big 4 CPA firm; or not in public accounting

^c^COCPA11 was a partner at a Big 4 accounting firm that declined to do a phone interview but responded via email that the firm’s policy is to not serve cannabis businesses because they are illegal at the federal level

### Data collection

Three central research questions were the focus of the study with sub-questions asked to narrow the focus as the interview was conducted. As interviews took place, responses were analyzed for clarity and understanding. At times, probes were asked to “follow up something already asked” and to seek additional information from the participants (Merriam 2009, p. 100). Data collection ceased at the point of data saturation, the point at which “the researcher is no longer hearing or seeing new information” (Astroth and Chung 2018; Savin-Baden and Major 2013, p. 317).

### Data analysis

The study utilized the five steps of the data analysis process from Creswell and Creswell (2018). First, the interview data were “organized and prepare [d]” for analysis by cataloging interviews by state and type, Certified Public Accountant or CRB owner, and copying, or cutting, into Excel according to interview question (Creswell and Creswell 2018, p. 193; Savin-Baden and Major 2013). Data was cleansed to eliminate conversation irrelevant to the research study. Interview data was reviewed to allow understanding of the overall ideas from the participants (Creswell and Creswell 2018). This immersion into the data helped to ensure a solid understanding prior to detailed analysis (Savin-Baden and Major 2013).

Interview transcripts were broken down into similar concepts or codes (Belotta 2018; Creswell and Creswell 2018). The codes were used to identify common themes in the data (Creswell and Creswell 2018). Where repetition was identified in the data, themes became apparent (Savin-Baden and Major 2013). Comparison, or triangulation, between themes identified in the CRB owner interviews and themes identified in the Certified Public Accountant interviews helped to ensure reliability of the research (Merriam 2009). Due to the small sample size, a Fisher’s exact test calculated using R statistical software was used to determine statistical significance when comparing responses between accountants that serve the industry and those that do not.

## Results

Several major themes emerged from the interview data. The primary reason given that Certified Public Accountants choose to not serve the industry is that cannabis remains illegal at the federal level. All ten of the CRB owners interviewed were able to find a Certified Public Accountant for tax services. The primary theme from CRBs and Certified Public Accountants who are serving the industry was the need for accountants to have a thorough understanding of the industry and IRS Tax Code Section 280E.

### Research question 1: why are some Certified Public Accountants unwilling to service the cannabis industry?

Eight of the Certified Public Accountant respondents indicated that the primary reason to avoid the industry was because the industry is federally illegal and/or they feared federal criminal prosecution. Four indicated that the special tax requirements and nuances with the tax code were not worth the extra technical training necessary to service what would be a niche industry. In addition, three worried that serving CRBs would jeopardize their Certified Public Accountant license. Table 4 shows the counts and percentages of Certified Public Accountants responding to the primary reasons they would not serve the cannabis industry.

**Table 4:** Reasons participating Certified Public Accountants would not serve the cannabis industry

Reason stated	Count (%) of Certified Public Accountant participants who do NOT serve the industry
Federally illegal; fear of criminal prosecution	8 (89%)^a^
Do not want to jeopardize Certified Public Accountant license	3 (33%)
Complicated tax requirements; not willing to devote the time and resources to special technical training;	4 (44%)
Banking/cash and security issues	1 (11%)

Certified Public Accountants may have given more than one reason to not serve the industry; therefore, percentages will not total 100%

^a^Includes COCPA11 that declined an interview but indicated his firm does not serve cannabis clients because it is illegal at the federal level

### Research question 2: how do CRBs compensate for lack of Certified Public Accountant services?

All ten of the CRB owners interviewed indicated that they currently engaged a Certified Public Accountant for their business, though two indicated that they previously had difficulty in finding a Certified Public Accountant that was willing to work with them and were competent. All ten of the CRBs had Certified Public Accountants that did their federal income tax filing. Five described their relationship with their Certified Public Accountant as being like an out-sourced CFO.

Five Certified Public Accountant participants indicated that they do serve the cannabis industry. Three of those five only serve cannabis clients and no other industries. Seven CRB participants and three Certified Public Accountants who currently serve the industry made a point to note that regardless of the service to be provided, Certified Public Accountants must have in-depth knowledge of the industry. Tax compliance was the service indicated as being needed most by CRBs from fifteen participants. Table 5 summarizes the responses when asked what professional accounting and tax services are most needed in the industry.

**Table 5:** Professional accounting and tax services perceived as most needed by participating cannabis-related businesses and Certified Public Accountants

Accounting and tax services most needed by the cannabis industry	Reported by participant type		
	Cannabis-related business owners count (%)	Certified Public Accountants that serve industry	Certified Public Accountants that do not serve industry
Certified Public Accountant must know industry in detail regardless of services provided	7 (70%)	3 (60%)	0
Tax compliance	5 (50%)	5 (100%)	5 (55%)
Accounting and tax advice	6 (60%)	3 (60%)	2 (22%)
Function like a CFO/Controller to help management make business decisions	5 (50%)		3 (33%)
Accounting and bookkeeping	0	3 (60%)	7 (77%)
Consulting on areas such as business valuations, M&A, internal controls	0	1 (20%)	5 (55%)
GAAP financial statement audits	0	1 (20%)	3 (33%)

Participants may have identified more than one needed service; therefore, percentages will not total 100%

### Research question 3: what does a Certified Public Accountant need to know prior to engaging to service the industry?

Table 6 summarizes the Certified Public Accountants’ responses to what is the greatest risk associated with serving the industry. Risk of federal prosecution and loss of Certified Public Accountant license were the two most frequently indicated responses. Certified Public Accountants who do not serve the industry identified more risks than those who do serve the industry (*p* = 0.04).

**Table 6:** Risk in serving the cannabis industry as reported by participating Certified Public Accountants

Risk for Certified Public Accountant serving the industry	Reported by participant type		
	Certified Public Accountants that serve industry	Certified Public Accountants that do not serve industry	Total Certified Public Accountants count (%)
Nothing different from any other client, we are helping them stay in compliance with federal tax law	2 (40%)	0	2 (14%)
Tax Code section 280E/Tax issues/Tax audits	1 (20%)	1 (11%)	2 (14)%
Relying on improper records to support income tax return	1 (20%)	1 (11%)	2 (14%)
Client may be doing something outside of regulations	1 (20%)	0	1 (7%)
Difficult to recruit staff	1 (20%)	0	1 (7%)
Getting sued/litigation	1 (20%)	1 (11%)	2 (14%)
Reputational risk	0	2 (22%)	2 (14%)
Losing Certified Public Accountant license	0	4 (44%)	4 (29%)
Federal prosecution	0	4 (44%)	4 (29%)
Cash-intensive business/Security of staff/Depositing cash receipts from CRBs	0	2 (22%)	2 (14%)
Lack of technical training on industry	0	2 (22%)	2 (14%)

Certified Public Accountants may have identified more than one risk factor; therefore, percentages will not total 100%

All participants were asked what banking and cash handling issues were unique to the cannabis industry. Ten participants expressed knowledge that most banks will not serve the industry and that those that do charge high fees. Eight participants acknowledged security issues related to having a cash-intensive business. Seven of ten CRBs indicated they had accounts closed at multiple banks and had to find new ones. Four CRBs in remote areas often had to transport cash long distances to a bank that would give them an account. Four CRBs identified alternative ways to bank including setting up management accounts to handle the cash through a bank, though these often were closed once the bank discovered the transactions were from a cannabis business. Table 7 shows these responses.

**Table 7:** Banking and cash difficulties for the industry as reported by cannabis-related business owners

Participant ID^a^	Banking and cash difficulties
WACRB1	• Bank is long-distance drive
	• Safety/security issues
	• Have had accounts closed
	• High fees
	• No credit cards
WACRB2	• Have had 2–3 accounts closed
	• Significant reporting requirements/bank audits
WACRB3	• Bank is long-distance drive
	• High fees
	• Difficult to keep cash records
	• Closed bank account and went back to all cash
WACRB4	• High fees
	• Safety/security issues
	• Have had 3–4 accounts closed
	• Use multiple accounts/management company for bank account
WACRB5	• Have had 3 accounts closed
	• Pay as much in cash as possible
	• Save up $20s and drive them to pay IRS in cash
	• Use management company for bank account
COCRB1	• Have had multiple accounts closed
	• Bank is long-distance drive
	• Hard to pay taxes without bank account
COCRB2	• High fees
	• Significant reporting requirements
	• Bank is long-distance drive
	• Safety/security issues
COCRB3	• High fees
	• Significant reporting requirements
	• Have had multiple accounts closed (even employees’ personal accounts were closed)
COCRB4	• Have had account closed
	• High fees
	• Use multiple accounts/management company for bank account
COCRB5	• High fees
	• Pay as much in cash as possible
	• Difficult to keep cash records
	• Bank “ignores” the type of business/turns a blind eye

^a^First two digits of participant ID represent the state, next three digits indicate whether the participant was a cannabis-related business owner (CRB) or Certified Public Accountant (CPA), and the last digit represents the number of the interviewee

Three Certified Public Accountants that work with the industry and three CRBs also indicated that there was complicated and time-consuming reporting required for cannabis bank accounts. Table 8 reports the responses by Certified Public Accountants regarding banking and cash issues compared to the responses given by the CRBs. There is not a statistically significant difference between the banking and cash issues identified by accountants that do serve the industry and those that do not (*p* = 0.35).

**Table 8:** Banking and cash issues reported by cannabis-related business owners (CRBs) compared to Certified Public Accountants

Banking and cash issues reported	Reported by participant type		
	Cannabis-related business owners count (%)	Certified Public Accountants that serve industry	Certified Public Accountants that do not serve industry
Banking issues vary by state	0	2 (40%)	0
High fees	7 (70%)	3 (60%)	0
Significant reporting requirements for bank	3 (30%)	3 (60%	0
Safety/security issues	3 (30%)	1 (20%)	4 (44%)
Some CRBs have bank accounts that are not openly cannabis accounts	4 (40%)	1 (20%)	0
No credit cards	1 (10%)	2 (40%)	0
Insider theft/susceptible to fraud	0	1 (20%)	1 (11%)
No banking available	0	0	4 (44%)
Complicated cash record-keeping	2 (20%)	0	2 (22%)
Bank is long distance drive	4 (40%)	0	0
Accounts get closed by bank	7 (70%)	0	0

Participants may have identified more than one banking and cash issue; therefore, percentages will not total 100%

All participants were asked what type of internal controls were needed in the industry to safeguard cash and inventory and ensure adherence to federal, state, and local regulations. Respondents described physical security controls eighteen times. CRB and Certified Public Accountant participants mentioned internal controls for record-keeping requirements needed to support both accounting records and meet state and local regulatory requirements twenty times in the various interviews. There is not a statistically significant difference between internal controls identified by accountants that do serve the industry and those that do not (*p* = 0.60). Table 9 reports the internal control needs described by interview participants.

**Table 9:** Internal controls needed to safeguard assets and ensure adherence to regulations in the cannabis industry

Internal controls needed	Reported by participant type		
	Cannabis-related business owners count (%)	Certified Public Accountants that serve industry	Certified Public Accountants that do not serve industry
Physical security controls			
Cameras	5 (50%)	1 (20%)	2 (22%)
Safes/Vaults	5 (50%)	0	0
Locks/Fencing/Guards	3 (30%)	1 (20%)	1 (11%)
Support record-keeping requirements			
Cash logs/records/reconciliation	2 (20%)	0	3 (33%)
Frequent inventory counts/reconciliation	2 (20%)	2 (40%)	3 (33%)
Technology to track inventory and sales	3 (30%)	3 (60%)	2 (22%)
Segregation of duties with cash/inventory handling	3 (30%)	2 (40%)	2 (22%)
No one alone with cash	0	1 (20%)	1 (22%)
Drug test employees	0	0	1 (11%)
Review/oversight of classification of costs for Tax Code section 280E	0	0	1 (11%)

Participants may have identified more than internal control; therefore, percentages will not total 100%

All Certified Public Accountant participants were asked what special training or technical knowledge is needed to serve the industry. The most common responses indicated that a solid understanding of the industry including how business operates, state and local regulations that must be followed, government and bank reporting requirements, and detailed knowledge of Tax Code Section 280E are critical. Table 10 reports the special training and technical knowledge Certified Public Accountants believe is needed to serve the industry competently. While there is not a statistically significant difference between the training and knowledge identified by accountants that do serve the industry and those that do not, it might be significant if there were more data as the *p* value is just slightly over 0.05 (*p* = 0.051). Table 11 lists quotes from participants regarding the need for Certified Public Accountants to have specialized knowledge of the industry.

**Table 10:** Special training and technical knowledge needed for a Certified Public Accountant to serve cannabis industry

Special training and technical knowledge	Reported by participant type	
	Certified Public Accountants that serve industry count (%)	Certified Public Accountants that do not serve industry
Knowledge of Tax Code Section 280E	4 (80%)	3 (33%)
Thorough knowledge of cannabis industry	4 (80%)	6 (66%)
Read everything available about industry	2 (40%)	0
Continuous learning needed	1 (20%)	1 (11%)
Understanding of cash-intensive business and internal controls	0	1 (11%)
Understanding of the technology used to track cash and inventory	0	1 (11%)
Nothing	0	1 (11%)

Certified Public Accountants may have identified more than one training factor; therefore, percentages will not total 100%

**Table 11:** Participant quotes regarding the need for significant cannabis industry knowledge by Certified Public Accountants

Participant ID^a^	Quote
COCPA2	“There’s a lot of nuance to [the industry] … where they’re allowed to deduct certain things, not allowed to deduct certain things. … [A Certified Public Accountant needs] some experience dealing with the companies themselves. They’ve got challenges.”
COCPA5	“The internal controls, to me, get stepped up to a whole new level when you’re talking about [the cannabis industry]. And so ramping up, at every aspect, whoever’s touching and interfacing with the client, if it’s an audit or most likely tax or advisory … whoever the team is, making sure they’re well versed in internal controls [for a cannabis business].”
COCPA6	“Really becoming savvy and understanding the industry would be key.”
COCPA7	“You certainly need to at least be familiar with the business model and industry. … So really understanding how that business is structured and how it works, and how the industry is structured and how it works is very important.”
COCPA8	“I don't think you can jump into it, not knowing what you're getting into, like even as a bookkeeper. I mean if you got into it, not knowing about 280E, you would drown. You would drown.”
WACPA1	“Knowledge of the industry is going to be really, really important.”
WACPA2	“You should read that code [280E]. … Here’s a couple IRS memos … that you need to read … here’s some Tax Court cases you need to read … and they have to understand that like, sort of the sprawling effect of all this 280E stuff is actually a lot more complicated than just cost of goods sold.”
WACPA3	“If you’re going to be in a niche, you really need to understand that business. You need to understand the industry and kind of each of the segments and how they’re interrelated and relate to and interact with each other.”
WACRB1	“I think that having a professional Certified Public Accountant that’s very familiar with cannabis law to do your end of the year tax return is very important.”
WACRB2	“Oh my God, somebody that knows something about the industry. … You got to know the industry. You got to know about compliance.”
WACRB3	“ … having that expertise and that knowledge and that confidence that definitely most businesspeople don’t have, and even [I] … didn’t have.”
COCRB1	“I would say that if you’re going to work with a traditional bookkeeper, it could be a little challenging because of 280E.”
COCRB3	CRBs need a Certified Public Accountant that “provides good 280E advice … know the industry, and 280E and IRS arguments inside and out.”
COCRB4	We “need a high-level Certified Public Accountant that knows the industry and has a good connection with an attorney that knows the industry. A Certified Public Accountant needs to be very hands-on in this business to serve as sort of an outsourced CFO to guide operating decisions around 280E.”

^a^First two digits of participant ID represent the state, next three digits indicate whether the participant was a cannabis-related business owner (CRB) or Certified Public Accountant (CPA), and the last digit represents the number of the interviewee

When asked to describe their cannabis client acceptance procedures, all five of the Certified Public Accountants working with the industry indicated that they try to get to know the client much like any other potential engagement. Table 12 summarizes the client acceptance procedures described by Certified Public Accountants that service the industry.

**Table 12:** Cannabis client acceptance procedures reportedly in use by Certified Public Accountants who currently serve the industry

Client acceptance procedures in use	Certified Public Accountants that serve industry count (%)
Make clear Tax Code Section 280E expectations/follow law/ethics	4 (80%)
Special language in engagement letter	2 (40%)
Require client to have a bank account	1 (20%)
Require retainer	1 (20%)
Not different from any other client	1 (20%)

Certified Public Accountants may have identified more than one procedure; therefore, percentages will not total 100%

## Discussion

This study provides a better understanding of the accounting and tax complexities of the cannabis industry as perceived by cannabis business owners and Certified Public Accountants who are already serving the industry. This study also provides explanation as to why Certified Public Accountants are reluctant or unwilling to serve the cannabis industry. This study has important implications for the public accounting profession as well as the cannabis industry.

When interviewing a Certified Public Accountant to provide accounting and tax services, there are several characteristics or qualifications that a CRB should consider. The primary consideration for hiring a Certified Public Accountant should be the accountant’s knowledge of the cannabis industry. The Certified Public Accountant needs to have more than just a cursory knowledge of the industry, but rather have an in-depth understanding. For example, the Certified Public Accountant should have a thorough working knowledge of Tax Code Section 280E and internal control issues unique to the industry. The Certified Public Accountant should have attended specific industry training or completed continuing professional education (CPE) courses. The CRB should consider how much CPE and how recently the Certified Public Accountant attended the CPE. The CRB should consider how many other cannabis clients the accountant already serves or will take on. Finally, the CRB may want to consider how the Certified Public Accountant will be able to assist as the cannabis business matures. CRBs may look to their Certified Public Accountant to provide them financial guidance much like an out-sourced CFO.

A Certified Public Accountant who is thinking about serving the cannabis industry needs to first understand the risks associated with that industry. The Certified Public Accountant participants already serving the cannabis industry did not indicate any particular themes related to risks of serving the industry. In fact, some felt there were no risks that were unique to serving this industry as opposed to others. However, the Certified Public Accountant participants not serving the cannabis industry indicated risks as potential federal prosecution and loss of the Certified Public Accountant license and also identified more risks than those accountants who do serve the industry. It is possible that accountants not serving the cannabis industry may have misconceptions about the industry influencing the risks they identify, while accountants serving the industry have first-hand knowledge of what the industry is like and where they experience risk versus reward. Last, a Certified Public Accountant should evaluate the need for specialized technical knowledge in tax and accounting for the cannabis industry.

While important with any potential client, client acceptance and continuance procedures must be completed carefully with a potential CRB client. The Certified Public Accountant should start with an interview to get to know the client. The Certified Public Accountant should thoroughly investigate the status of the cannabis license or license application with the state to determine the legality of the business within state and local regulations. The Certified Public Accountant should make the potential client aware of the expectations regarding compliance with all laws and regulations with specific emphasis on 280E and tax compliance. The Certified Public Accountant may wish to learn more about the CRB owner by talking with other business contacts and references. None of the Certified Public Accountant participants in this study conducted background checks for CRB clients, but this could be done if the Certified Public Accountant had concerns. A Certified Public Accountant could also consider collecting a retainer from the CRB client. Last, the Certified Public Accountant could modify the engagement letter to clarify expectations regarding staying in compliance with all state and local regulations and federal tax code.

A Certified Public Accountant entering the cannabis industry should expect continuous learning for the industry. First, the Certified Public Accountant should check with the state board of accountancy to determine if there is any guidance for Certified Public Accountants in that state related to the cannabis industry. Next, the Certified Public Accountant needs to read Code Section 280E in its entirety. The accountant also needs to read all Tax Court cases related to the cannabis industry and understand that the cases thus far have overwhelmingly found against the cannabis businesses. The Certified Public Accountant needs to continually read about new tax cases and other current events related to the industry. There are many CPE courses available to Certified Public Accountants that are specific to the cannabis industry. The Certified Public Accountant should consult the AICPA, their state society of Certified Public Accountants, or look to more mature cannabis states for learning opportunities. For example, the Colorado Society of Certified Public Accountants holds an annual symposium for Certified Public Accountants interested in the cannabis industry. In addition, the accountant may want to interview a Certified Public Accountant from a more mature state to get advice on how to competently proceed and minimize risk.

The Certified Public Accountant should remember that the nuances of accounting for the cannabis industry go beyond Tax Code Section 280E. For example, in newer cannabis states, the CRB may not be able to find a bank account. The Certified Public Accountant needs to be prepared to guide the CRB in establishing effective internal controls that will enable the CRB to safeguard inventory and cash, comply with all state and local regulations, and ensure reliable financial record-keeping of mostly cash transactions. The required internal controls will include, but go far beyond physical controls such as cameras, vaults, and locks as required by regulations. State regulations also require effective and detailed inventory tracking, usually from seed to sale. Internal controls will also need to be sufficient to not only safeguard cash and cannabis inventory from theft but also ensure accurate revenue and expense records in sufficient detail for the Certified Public Accountant to properly complete tax returns at the federal, state, and local levels. Once banking is available to the CRB, a new set of reporting requirements will need to be met to maintain the account. Many CRBs will look to their Certified Public Accountant for help with these internal controls and reporting requirements.

Only one of the Certified Public Accountant participants from this study provides audit or other attest services to the cannabis industry. In general, there are many Certified Public Accountants who do not provide any audit or attest services to any industry due to litigation risks. Some states, like Colorado, may at their discretion require a financial statement audit of a CRB by a licensed Certified Public Accountant. In addition, as the cannabis industry evolves, there may be additional funding opportunities which would put cannabis companies on the securities market as has already taken place in Canada. This would cause these cannabis companies to require annual audited financial statements from a licensed Certified Public Accountant. While this study found there are Certified Public Accountants that specialize in providing, or are at least willing to provide, accounting and tax services to the cannabis industry, there are not many firms that will do an audit for the industry. This presents additional opportunity for Certified Public Accountants in the cannabis marketplace.

### Limitations

This study has several limitations including limited external validity and potential selection bias. The views of the interviewees may not necessarily generalize to all CRB owners or Certified Public Accountants in these two states or other states. However, repetition of themes indicating data saturation is a good indication that the responses would be similar if more CRB owners or Certified Public Accountants were interviewed. Finding participants for the study was difficult as many of those invited declined to participate. The convenience sample drawn could have introduced some level of selection bias into the study. Those who declined participation may have had different views of the industry than those that agreed to participate. In addition, interview responses may be influenced by the interviewer’s presence and some participants may not be “equally articulate and perceptive” (Creswell and Creswell 2018, p. 188). While questions were asked in a neutral tone and data was analyzed without intentional bias, unconscious bias could be present in the study.

### Recommendations for future research

This study could be expanded to additional states to broaden this field of knowledge. Interviews of participants from states that are newer to the cannabis marketplace may result in findings that are different from the findings of this study of two mature cannabis states. A future study could compare the findings from mature states and developing states. Another interesting follow up study could investigate organization stigma issues for Certified Public Accounting firms working in the cannabis industry.

Quantitative studies could be designed to obtain data from a larger sample size than is possible with a qualitative study. For example, Certified Public Accountants could be surveyed to determine their level of agreement with the theory identified herein that the greatest risk in Certified Public Accountants providing services to CRBs is the risk of federal prosecution. CRB owners could be surveyed to determine their level of agreement with the theory that they need Certified Public Accountants who have in-depth knowledge of the industry and would be able to perform as an outsourced CFO to guide their business into the future. Another quantitative study could be conducted to further explore the motivational or ethical factors as to why Certified Public Accountants choose to serve this industry or not. A follow up quantitative study has already begun in which Certified Public Accountants have been surveyed to determine their level of agreement with the risks identified in this project and whether they perceive any ethical issues in working with the industry.

## Conclusion

The cannabis industry remains in limbo with state laws in conflict with federal law. The findings of this study indicate that, while many Certified Public Accountants will not serve the cannabis industry, there are competent and knowledgeable Certified Public Accountants who will. CRB owners need to carefully consider the industry knowledge and experience of a potential Certified Public Accountant prior to engaging them.

For Certified Public Accountants that are considering accepting CRB clients, this study showed that there are risks that should be weighed such as the risk of federal prosecution and potential loss of the Certified Public Accountant license. Next, the Certified Public Accountant must commit to acquiring and maintaining substantial specialized knowledge related to tax Code Section 280E, internal controls for a cash-only or cash-intensive business, and the workings of the cannabis industry under the current regulatory conditions. As cannabis legalization continues to expand to new states, the need for Certified Public Accountants to serve the industry continues to grow. This creates considerable business opportunity for Certified Public Accountants who are willing to bear the related risk.

## Supplementary Information

**Additional file 1.** Appendix A.

**Additional file 2.** Appendix B.

## Data Availability

Interview transcripts and manual notes as well as Excel files summarizing data were maintained.

## Abbreviations

CRB: Cannabis-related business
CPA: Certified Public Accountant
BOA: Board of Accountancy
