# Supplementary material for: Accounting and the US cannabis industry: federal financial regulations and the perspectives of Certified Public Accountants and cannabis businesses owners
Source: J Cannabis Res. 2020 Dec 3;2:41. doi: 10.1186/s42238-020-00049-7 (PMC7819304; doi:10.1186/s42238-020-00049-7)
Supplement: Supplementary file 1 — Additional file 1. Appendix A. [file 42238_2020_49_MOESM1_ESM.docx]

*Appendix A*

Interview Questions for CPAs

Following are the questions for interviews of Certified Public Accountants:

1. Demographics
2. In what state(s) do you hold a CPA license?

□ CO

□ WA

□ Other: Please list

1. In what zip code do you primarily practice?

_ _ _ _ _

1. Which of the following best describes your CPA firm?

□ Single CPA

□ Local CPA firm with multiple CPAs

□ Regional CPA firm

□ National CPA firm

□ Big 4 CPA firm

□ Not in public accounting

1. Do you or your firm provide services to a marijuana-related business (MRB)? If yes, go to question 3. If no, skip to question 12.

□ Yes (go to question 3)

□ No (go to question 12)

1. What types of services do you provide to MRBs? Select all that apply:

□ Tax

□ Bookkeeping

□ Audit

□ Consulting (describe)_______________________________________________

□ Other (describe)___________________________________________________

1. Describe the types of client acceptance procedures you perform prior to accepting the MRB client?
2. What type of special training or technical knowledge do you believe is required to service the MRB?
3. Are there any services that you choose NOT to provide to MRBs? Why/why not?
4. What do you believe is the greatest potential risk related to providing services to MRBs?
5. What type of banking and cash handling difficulties, if any, do you believe are unique to the marijuana industry?
6. Describe the internal controls to safeguard cash and inventory and ensure adherence to federal, state and local regulations that you believe are unique to the marijuana industry.
7. Describe the professional accounting or tax services that you believe are needed most for marijuana businesses.

END INTERVIEW FOR THOSE WHO ANSWERED YES ON NUMBER 2

REMAINDER OF INTERVIEW FOR THOSE WHO ANSWERED NO ON NUMBER 2

1. Why do you not provide service to MRBs?
2. What would most likely persuade you to take on MRB clients?
3. What type of special training or technical knowledge do you believe is required to service the MRB?
4. What do you believe is the greatest potential risk related to providing services to MRBs?
5. What type of banking and cash handling difficulties, if any, do you believe are unique to the marijuana industry?
6. Describe the internal controls to safeguard cash and inventory and ensure adherence to federal, state and local regulations that you believe are unique to the marijuana industry.
7. Describe the professional accounting or tax services that you believe are needed most for marijuana businesses.
