# Supplementary material for: Accounting and the US cannabis industry: federal financial regulations and the perspectives of Certified Public Accountants and cannabis businesses owners
Source: J Cannabis Res. 2020 Dec 3;2:41. doi: 10.1186/s42238-020-00049-7 (PMC7819304; doi:10.1186/s42238-020-00049-7)
Supplement: Supplementary file 2 — Additional file 2. Appendix B. [file 42238_2020_49_MOESM2_ESM.docx]

*Appendix B*

Interview Questions for MRB Owners

Following are the questions for interviews of marijuana-related business owners:

1. Describe your marijuana-related business (MRB) in terms of type, approximate yearly revenues, legal structure, year established.
2. What type of banking and cash handling difficulties, if any, do you have that are unique to the marijuana industry?
3. Do you engage a CPA to provide services for your marijuana business? If yes, see #4. If no, see #5.
4. Describe your business relationship with your CPA in terms of services provided and terms of engagement.
5. How do you substitute for CPA services?
6. Describe your internal controls to safeguard cash and inventory and ensure adherence to federal, state and local regulations. (Internal controls are the policies and procedures put into place to safeguard assets, ensure reliable financial reporting, ensure adherence to laws and regulations, and promote operational efficiency.)
7. Has your business been audited by the IRS?
8. Describe the professional accounting or tax services that are needed most for your marijuana business.
